# Supplementary material for: Drug mechanism enrichment analysis improves prioritization of therapeutics for repurposing
Source: BMC Bioinformatics. 2023 May 24;24:215. doi: 10.1186/s12859-023-05343-8 (PMC10207828; doi:10.1186/s12859-023-05343-8)
Supplement: Supplementary file 1 — Additional file 1. Supplemental Figures 1-5. [file 12859_2023_5343_MOESM1_ESM.docx]

**Supplemental Figures**

**Drug mechanism enrichment analysis improves prioritization of therapeutics for repurposing**

Belinda B. Garana^1^, James H. Joly^1,†^, Alireza Delfarah^1,‡^, Hyunjun Hong^2^, and Nicholas A. Graham^1,3,4^

^1^Mork Family Department of Chemical Engineering and Materials Science, University of Southern California, Los Angeles, CA, USA

^2^Department of Computer Science, Information Systems, and Applications, Los Angeles City College, Los Angeles, CA, USA

^3^Norris Comprehensive Cancer Center, University of Southern California, Los Angeles, CA, USA

^4^Leonard Davis School of Gerontology, University of Southern California, Los Angeles, CA, USA

^†^Current address: Nautilus Biotechnology, San Carlos, CA, USA

^‡^Current address: Calico Life Sciences, South San Francisco, CA

To whom correspondence should be addressed:

Nicholas A. Graham, University of Southern California, 3710 McClintock Ave., RTH 509, Los Angeles, CA 90089. Phone: 213-240-0449; E-mail: [nagraham@usc.edu](mailto:nagraham@usc.edu)

## DATA AVAILABILITY

All code and data used in this manuscript, as well as a web application and an R package to run DMEA, are available at <https://belindabgarana.github.io/DMEA>.

## Supplementary Figures


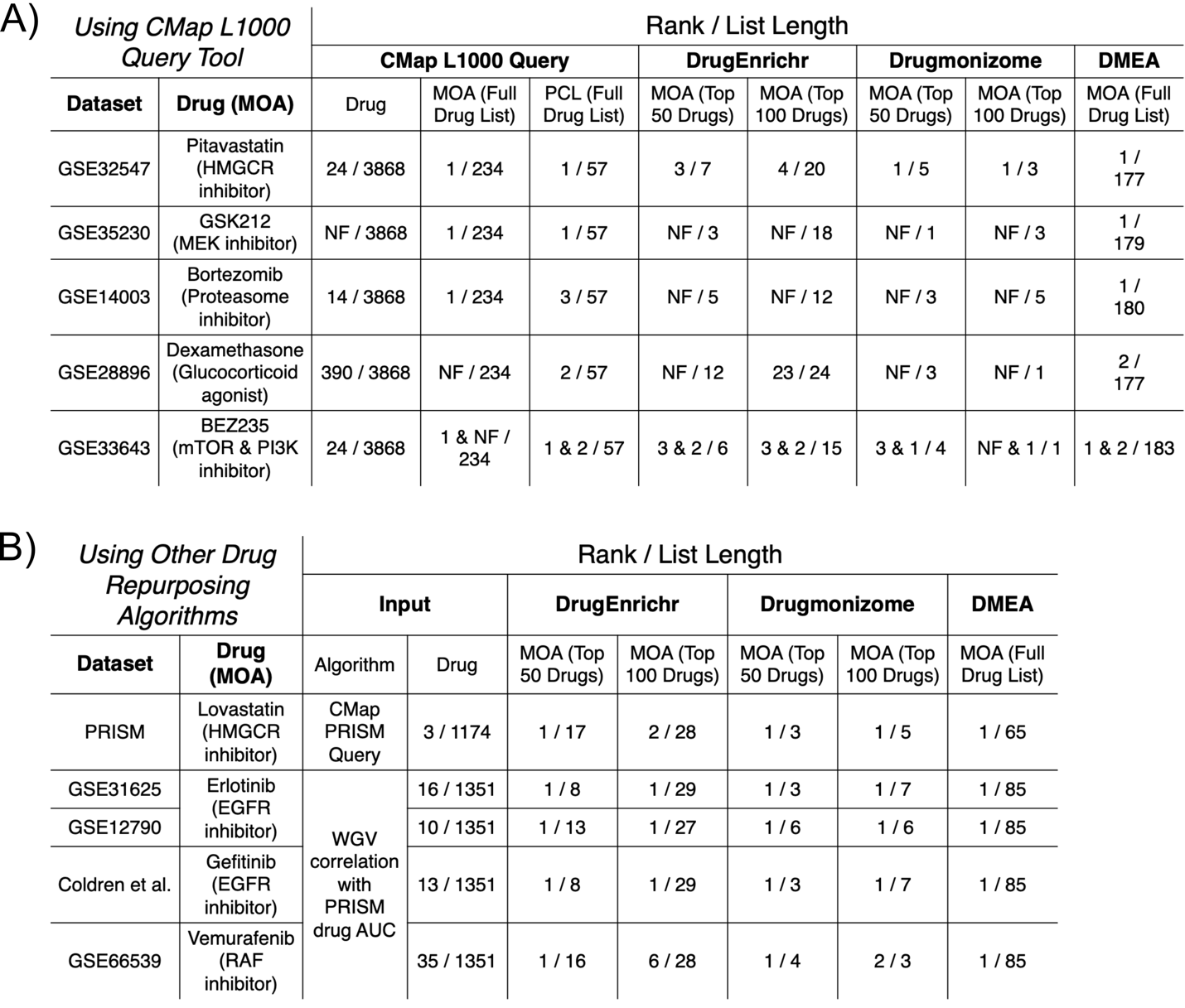


**Supp. Figure 1. DMEA generates MOA rankings that are improved over single-drug rankings and similar to or better than other tools’ rankings of drug MOA.** A) Comparison of DMEA’s MOA rankings to rankings generated by the CMap L1000 Query, DrugEnrichr, and Drugmonizome. DMEA’s rankings are improved compared to single-drug rankings and similar or improved compared to other tools for evaluating drug MOAs in all cases. NF: not found. B) DMEA’s MOA rankings also improve upon single-drug rankings in other cases where CMap cannot provide MOA or PCL analysis and are similar compared to DrugEnrichr and Drugmonizome which only evaluate a relatively small input list of drug names.


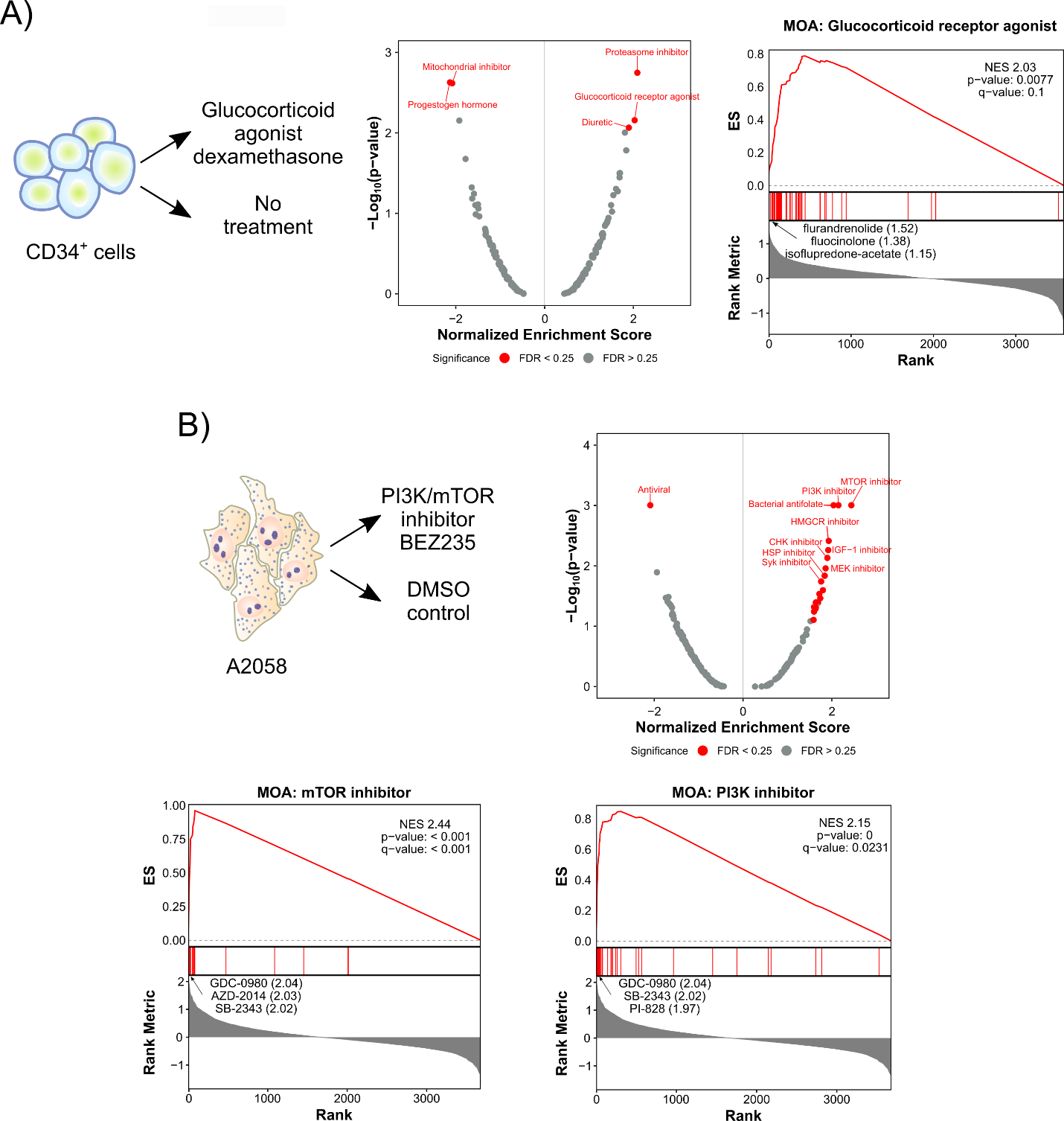


**Supp. Figure 2. DMEA identifies similar MOAs based on gene expression connectivity scores.** Rank-ordered drug lists were generated by querying the CMap L1000 gene expression perturbation signatures and then analyzed by DMEA. A) Human CD34+ cells treated with the glucocorticoid agonist dexamethasone (24 h) or untreated [1]; B) A2058 cells treated with the PI3K/MTOR inhibitor BEZ235 or DMSO [2]*.* Volcano plots summarizing the NES and -log­_10_­(p-value) for all tested drug MOAs and mountain plots of the expected MOAs are shown. Red text indicates MOAs with p-value < 0.05 and FDR < 0.25.


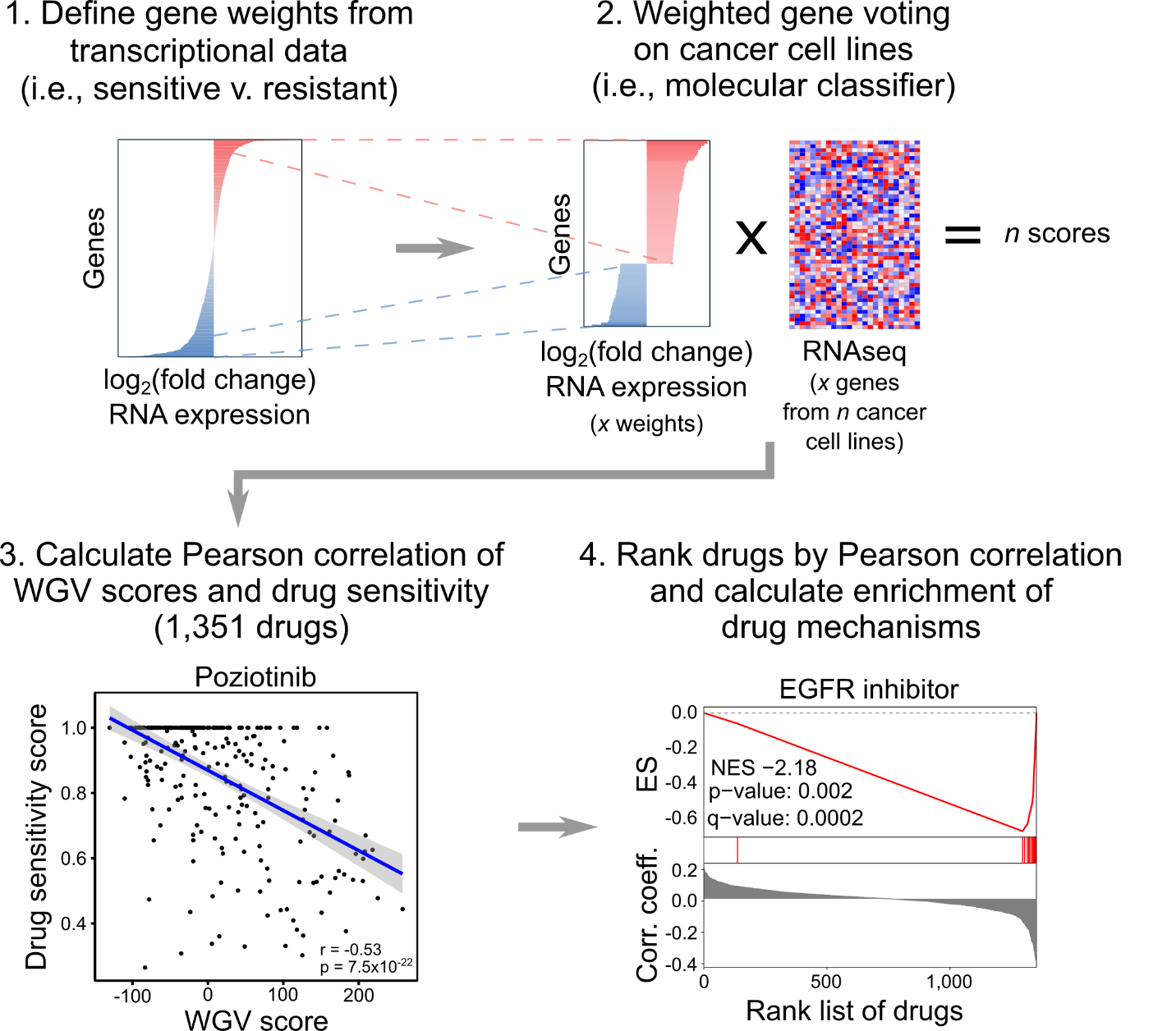


**Supp. Figure 3. Overview of drug mechanism enrichment analysis using WGV molecular classification scores.** DMEA can accept an input gene signature when paired with a molecular classification method such as weighted gene voting (WGV), correlations, and large public databases of gene expression and drug screens. First, gene weights are calculated as log_2_(fold-change) values calculated between two groups of samples from transcriptional data. Only the *x* genes with log_2_(fold-change) with q-value < 0.05 are selected for subsequent steps (maximum of 500 genes). Second, the WGV molecular classifier score is calculated as the dot product between *x* gene weights and gene expression values from cancer cell lines (*x* genes by *n* cell lines). Here, we used 327 adherent cancer cell lines from the CCLE. Third, the Pearson correlation is calculated between the WGV score and drug sensitivity score (i.e., area under the curve (AUC) of cell viability versus drug concentration from the PRISM database) for each of the 1,351 drugs. An example is shown for the EGFR inhibitor poziotinib. Fourth, the 1,351 drugs are ranked by Pearson correlation coefficient, and the ranked list is analyzed using a GSEA-like algorithm to identify drug mechanisms that are enriched at either end of the ranked list. In total, 85 mechanisms-of-action are tested (Supp. Table 1). An example for EGFR inhibitors is shown. Each red tick mark represents a drug annotated as an EGFR inhibitor in the waterfall plot of correlation coefficients.


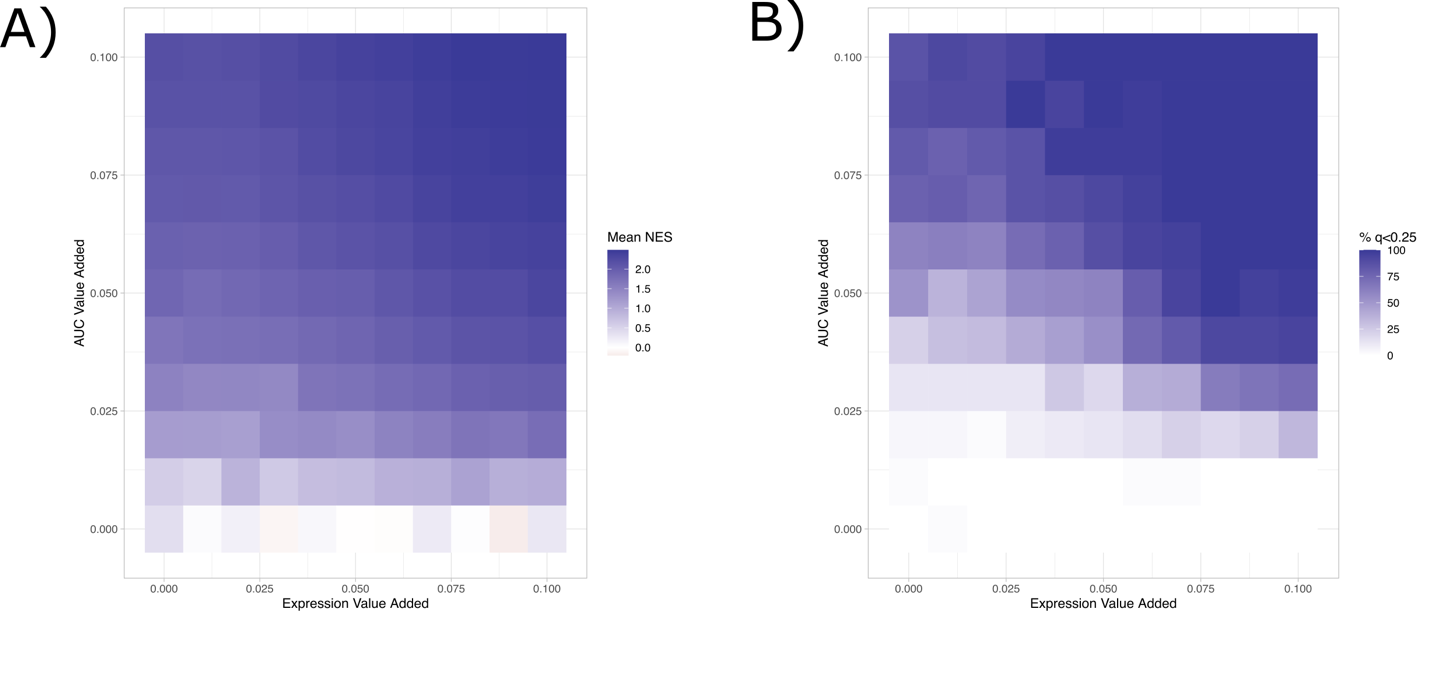


**Supp. Figure 4. Sensitivity analysis of DMEA pipeline with WGV using synthetic data.** Synthetic gene expression and drug sensitivity data was simulated with varying perturbations to RNA expression (x-axis) and drug sensitivity score (i.e., AUC, y-axis) (see Methods). A) Heatmap showing the average DMEA NES from 50 simulations for varying perturbations of the drug AUC and gene expression data. B) Heatmap showing the percent of DMEA replicates with FDR q-value < 0.25 from 50 simulations for varying perturbations of the drug AUC and gene expression data.


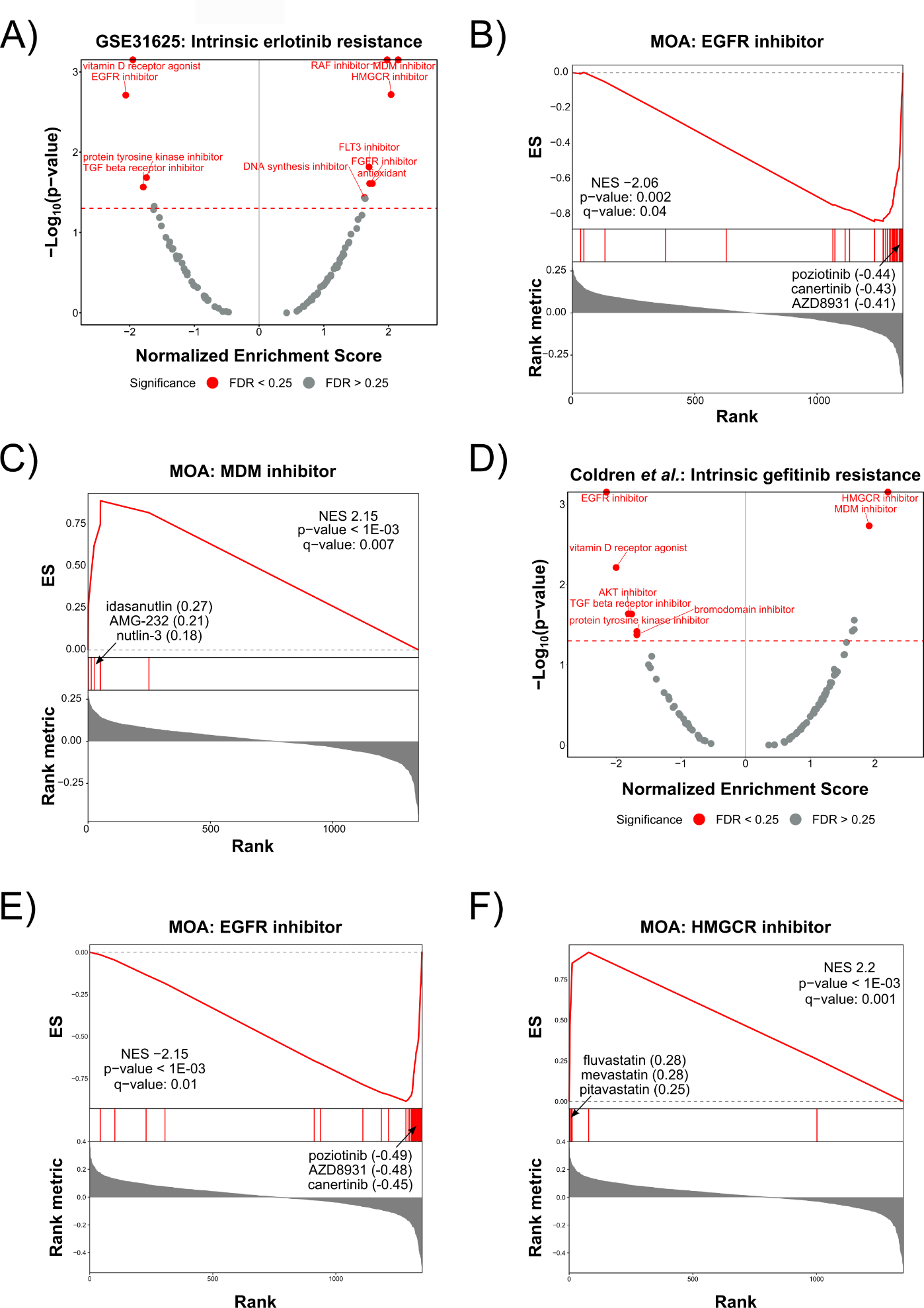


**Supp. Figure 5. DMEA identifies enrichment of the EGFR inhibitor MOA and other MOAs using external signatures of intrinsic EGFR inhibitor resistance.** Using gene expression signatures of intrinsic erlotinib (GSE31625 [3]) and gefitinib resistance (Coldren et al*.* [4]), we calculated WGV scores for 327 adherent cancer cell lines in the CCLE database. For each signature, the WGV scores were correlated with drug sensitivity data (i.e., AUC) for 1,351 drugs from the PRISM database. Drugs were then ranked by correlation coefficient, and DMEA was performed to identify enriched MOAs at either end of the rank-ordered drug list. A) Volcano plot of NES versus -log_10_(p-value) for DMEA using the GSE31625 signature of erlotinib resistance. Red text indicates MOAs with p-value < 0.05 and FDR < 0.25. B) Mountain plot showing that DMEA identified the EGFR inhibitor MOA as negatively enriched in the rank-ordered drug list generated using the GSE31625 signature of erlotinib resistance. The most negatively correlated EGFR inhibitors are highlighted along with their correlation coefficients. C) Mountain plot showing that DMEA identified the MDM inhibitor MOA as positively enriched in the rank-ordered drug list generated using the GSE31625 signature of erlotinib resistance. The most positively correlated MDM inhibitors are highlighted along with their correlation coefficients. D) Volcano plot of NES versus -log_10_(p-value) for DMEA using the Coldren et al. signature of gefitinib resistance. Red text indicates MOAs with p-value < 0.05 and FDR < 0.25. E) Mountain plot showing that DMEA identified the EGFR inhibitor MOA as negatively enriched in the rank-ordered drug list generated using the Coldren et al. signature of gefitinib resistance. The most negatively correlated EGFR inhibitors are highlighted along with their correlation coefficients. F) Mountain plot showing that DMEA identified the HMGCR inhibitor MOA as positively enriched in the rank-ordered drug list generated using the Coldren et al. signature of gefitinib resistance. The most positively correlated HMGCR inhibitors are highlighted along with their correlation coefficients.
